# Corrigendum to ‘Understanding p53 functions through p53 antibodies’

**DOI:** 10.1093/jmcb/mjz110

**Published:** 2019-12-26

**Authors:** Kanaga Sabapathy, David P Lane

**Affiliations:** 1 Laboratory of Molecular Carcinogenesis, Division of Cellular & Molecular Research, Humphrey Oei Institute of Cancer Research, National Cancer Centre Singapore, 11 Hospital Drive, Singapore 169610, Singapore; 2 Cancer and Stem Cell Biology Program, Duke–NUS Graduate Medical School, 8 College Road, Singapore 169857, Singapore; 3 Department of Biochemistry, National University of Singapore (NUS), 8 Medical Drive, Singapore 117597, Singapore; 4 Institute of Molecular and Cellular Biology, 61 Biopolis Drive, Singapore 138673, Singapore; 5 p53 Laboratory (p53Lab), Agency for Science, Technology, and Research (A*STAR), Singapore 138648, Singapore

In this article, we have inadvertently omitted the support from the funding agency, which should be as follows:

